# Anxiety among Pregnant Women about Corona Virus Infections during COVID-19 Pandemic at a Tertiary Care Center in Nepal: A Descriptive Cross-sectional Study

**DOI:** 10.31729/jnma.5377

**Published:** 2021-02-28

**Authors:** Dipty Shrestha, Rachana Saha, Naresh Manandhar, Asmita Adhikari, Jyoti Dahal

**Affiliations:** 1 Department of Obstetrics and Gynaecology, Kathmandu Medical College and Teaching Hospital, Sinamangal, Kathmandu, Nepal; 2 Department of Community Medicine, Kathmandu Medical and Teaching Hospital, Sinamangal, Kathmandu, Nepal

**Keywords:** *anxiety*, *COVID-19*, *fear*, *pregnancy*

## Abstract

**Introduction:**

COVID-19 is a beta coronavirus that is transmitted by physical interaction or close contact. This Coronavirus Pandemic has also created stress and anxiety among pregnant women all over the world. The disease was first identified in Wuhan city, China, in late December 2019 and was declared pandemic by the World Health Organization on 11th March 2020. Concern and stress in pregnancy are associated with pre-eclampsia, intrauterine growth restriction, preterm labour, depression etc. Pregnancy is an immune-compromised state and poses a high risk to this risk. This study aims to identify anxiety about the coronavirus infection among pregnant women visiting a tertiary care centre in Kathmandu, Nepal, during this COVID-19 pandemic.

**Methods:**

This is a descriptive cross-sectional study conducted at Kathmandu Medical College and Teaching Hospital from 15th July 2020 to 30th July 2020 after taking the ethical clearance from the Institutional Review Committee of Kathmandu Medical College. Convenient sampling method was used. All the data were entered in Statistical Package for the Social Sciences data 20.0 and analyzed. Data was presented in frequencies, charts and percentage.

**Results:**

Among the total 273 cases, only 2 (0.73%) case had a score between 25-30 corresponding to moderate to severe anxiety, 21 (7.69%) had a score between 18-24, which corresponds to mild to moderate anxiety and 250 (91.57%) had score 0-17 which corresponds mild status.

**Conclusions:**

Most of the participants in the study reported a mild status of anxiety. Very few participants reported moderate to severe anxiety.

## INTRODUCTION

COVID-19 is an infectious disease that affects the respiratory tract and is caused by severe respiratory syndrome coronavirus 2 (SARS-COV-2) first identified in Wuhan, China, in December 2019.^1^ WHO declared this outbreak a pandemic on 11^th^ march 2020.^2^ The number of pregnant women with COVID-19 is also increasing, there were already more than 30 pregnant patients with COVID-19 in China by February 8, 2020.^3^ The virus has caused tremendous anxiety and fear about the continuation of pregnancy, effect on newborn and chances of vertical transmission.^4^ In these challenging times associated with COVID-19, when there are several social and environmental disruptions, the pregnant women might feel further anxious and fearful about their pregnancy, their own health and fetus.^5^

COVID-19 in pregnancy has become today's great concern which needs to be identified and taken care of to prevent major complications in future.

Thus, this study is aimed at identifying anxiety among pregnant women during this COVID-19 pandemic.

## METHODS

This study was a descriptive cross-sectional study conducted at Kathmandu Medical College from July 15^th^ 2020 July 30^th^ 2020. The pregnant women visiting KMCTH for obstetric services irrespective of gestational age were enrolled. Ethical clearance (IRC Reference No.207202002) was taken from the Institutional Review Committee of the same institution on 2^nd^ of July 2020. A total of 273 cases were taken into account. The study included all the pregnant women visiting KMCTH for obstetric services, excluding pregnant women with medical disorders like HTN, GDM, chronic illness, psychiatric problems, drug and alcohol abuse, family history of psychiatric disorders. All the cases in the study were enrolled by convenient sampling and were explained about the study and informed consent was taken.

Each case was interviewed in the Outpatient Department (OPD) and the Obstetric Ward using the Hamilton Anxiety Rating Scale (HAM-A) and a well-structured questionnaire. The questionnaire included demographic profile, general history, past and present obstetric history, history of past illness, family history, and other measures assessing the psychosocial stress like alcohol and drug abuse and domestic violence. The questionnaire, along with the Hamilton Anxiety Rating Scale (HAM-A), was used. Pregnant women fulfilling the criteria were enrolled in the OPD five times a week. Two days were my OPD, and in my non OPD days, my doctor colleagues were explained about the procedure, and they filled the questionnaire. The sample size was calculated as below;


$$\begin{array}{ll} \text{n} & ={\text{Z}}^{\text{2}}\times \text{p}\times \text{q}/{\text{e}}^{\text{2}}\\ & ={\left(1.96\right)}^{2}\times 0.5\times 0.5/{\left(0.06\right)}^{\text{2}}\\ & =267 \end{array}$$

n = sample size
p = prevalence of 50% for maximum sample size
q = 1-p
e = margin of error, 6%
Z = 1.96 at 95% CI

Hamilton Anxiety Rating Scale was used in this study to find the anxiety level in pregnant women. Thus scale is a well-structured scale used worldwide to assess the extensiveness of anxiety in pregnancy. It has been used in various other studies. It consists of 14 items that are given, scoring 0 to 4 with a total of 0-56. Score 17 indicates mild anxiety, 18-24 indicates mild to moderate and 25-30 indicates moderate to severe.

All the data were entered into SPSS data 20.0 and analyzed. Data was presented in frequencies, charts and percentage.

## RESULTS

**Figure 1.**
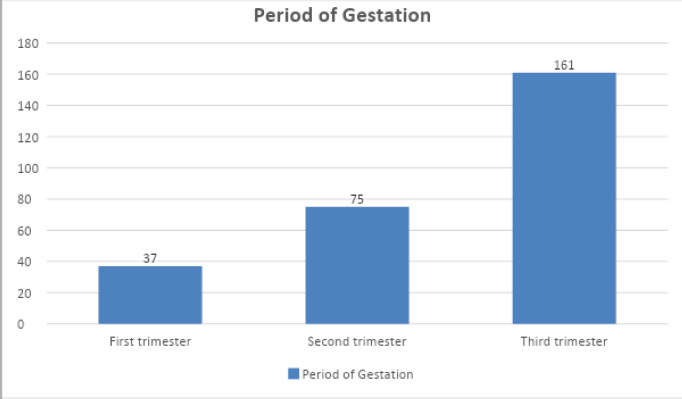
Distribution of the participants according to the period of gestation.

**Figure 2.**
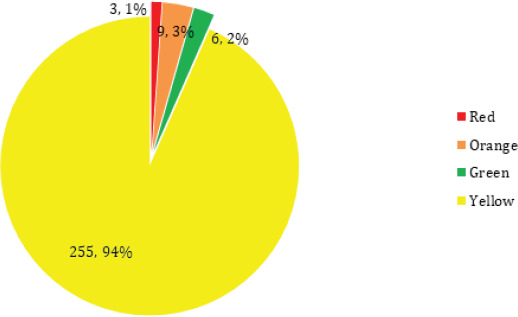
Distribution of the participants according to the category of the Zones of Nepal.

Among the total 273 cases, maximum of 161 (61.17%) were in the third trimester, 75 (27.47%) of them were in the second trimester and only 37 (13.55%) were in the first trimester (Figures 1 and 2).

Among the total 273 participants, only 2 (0.73%) had Hamilton Anxiety Scale of 25-30 corresponding to moderate to severe anxiety and maximum of them; 250 (91.57%) had mild anxiety (Tables 1, 2, and 3).

**Table 1:** Distribution of the participants according to the Anxiety Scale.

Hamilton Anxiety Scale	n (%)
<17	250 (91.57%)
17-24	21 (7.69%)
25-30	2 (0.73%)

**Table 2:** Period of gestation and Hamilton anxiety scale.

Trimester	Hamilton Anxiety Scale		
	< 17 n (%)	17-25 n (%)	25-30 n (%)
First	31 (83.78%)	6 (16.2%)	0
Second	68 (90.67%)	7 (9.33%)	0
Third	151 (93.79%)	8 (4.97%)	2 (1.24%)
Total	250 (91.57%)	21 (7.69%)	2 (0.732%)

**Table 3:** Parity and anxiety scale.

Gravida	Hamilton Anxiety Scale		
	< 17 n (%)	17-25 n (%)	25-30 n (%)
Primi	108 (39.56%)	6 (2.19%)	1 (0.87%)
Multi	142 (52.01%)	15 (5.49%)	1 (0.63%)
Total	250 (91.57%)	21 (7.69%)	2 (0.73%)

In this study, 8 (3.1%), 25 (9.3%), 17 (6.2%) and 255 (94%) of the cases were from the red zone, orange zone, green zone and the yellow zone, respectively.

## DISCUSSION

Today the entire world is battling against this powerful threat of COVID-19. One of the inevitable and sequelae consequences of this pandemic is the psychological impact on the vulnerable populations, such as pregnant women which needs to be addressed without delay. Indeed, pregnant women are known to be more emotionally vulnerable, to develop ambivalent feelings, or to have concerns about the future and/or about their ability to cope with the social demands of motherhood.^6-8^

This study was conducted with an effort to study the level of anxiety and fear in pregnancy. Pregnancy is a special time with excitement and various anticipation and expectations. Anxiety is a feeling of unease, worry or fear that can be mild to severe. Although anxiety and fear are common in pregnancy, in this study majority of them were in the mild category where among the total cases, 91.57% scored Hamilton score less than 17 accounting for mild status, 7.69% scored between 17-24, indicating mild to moderate anxiety and only 0.732% scored 25-30 indicating moderate to severe anxiety. In contrast to our study, a study by Corbett et al. showed that most pregnant women, 83.1%, did not worry about their health status before the pandemic of COVID-19, but during the pandemic 50-70% were worried about their health status most of the time.^9^

In a study by Ferit Durankus and Erson Aksu among pregnant women, 35.4% scored higher than 13 on the EPDS.^10^ A study by Gabriele Saccone et al. in China showed that more than half of the respondents (53%) rated the psychological impact as severe.^11^ As in our study, in this study, also more women were in the third trimester, in this study out of a total of 100 women, 17, 35 and 48 were in the first, second and third trimester, respectively.^11^

In this study, among the total 273 cases enrolled, most of them were in the age group of 25-29. The mean age of respondents was 27.7 years, with standard deviation was 4.415 years. The minimum age was 16 years, and the maximum age was 40 years. Among the total cases enrolled in this study, the majority were multigravida (58.2%) and (41.7%) were primigravida. In relation to the gestation period, 61.17% were in the third trimester, 27.47% were in the second trimester, and only 13.55% were in the first trimester. In the first trimester, the visit seems to be less than in the third trimester maybe because of the fear of COVID and fewer complications. In a similar study by Najmieh Saadati, women's mean age was 25.8±5.1, 27.2±5.7 and 26.5 ±4.5 in the first, second and third trimester, respectively.^12^ Similarly, in the same study, the total score of the anxiety was 22.3±9.5, 24.6±9.3 and 25.4±10.6 in the first, of the second and third trimester and totally 9%, 13% and 21% of the women had severe anxiety or scores >35 in the first, second and third trimester respectively.^12^ In another study by Nazil Hossain and et al. out of the total, 14.3% of women had score 7 or more and mean age of 26.47±4.8 years were enrolled, mean gestational age of women was 33.04±7.54 weeks.^5^

At the time of the study, zones of Nepal were categorized as red (very high risk), orange (high risk), yellow (moderate risk) and green (low risk) as per the cases and transmission. In this study,^3,9,6^ and 255 of the cases were from the red zone, orange zone, green zone and the yellow zone, respectively.

It is a small-scale study, and due to short time, long term outcomes could not be assessed. Data is not sufficient to support anxiety in pregnancy during this COVID 19 Pandemic

## CONCLUSIONS

In this study, anxiety in pregnant women is uncertain and requires further study due to the limited data. Most of the participants in the study reported a mild status of anxiety. Very few participants reported moderate to severe anxiety.
